# Deciphering the Complexity of 3D Chromatin Organization Driving Lymphopoiesis and Lymphoid Malignancies

**DOI:** 10.3389/fimmu.2021.669881

**Published:** 2021-05-14

**Authors:** Laurianne Scourzic, Eralda Salataj, Effie Apostolou

**Affiliations:** Sanford I. Weill Department of Medicine, Sandra and Edward Meyer Cancer Center, Weill Cornell Medicine, New York, NY, United States

**Keywords:** 3D chromatin organization, lymphopoiesis, activation, lymphoid malignancies, B cells, T cells

## Abstract

Proper lymphopoiesis and immune responses depend on the spatiotemporal control of multiple processes, including gene expression, DNA recombination and cell fate decisions. High-order 3D chromatin organization is increasingly appreciated as an important regulator of these processes and dysregulation of genomic architecture has been linked to various immune disorders, including lymphoid malignancies. In this review, we present the general principles of the 3D chromatin topology and its dynamic reorganization during various steps of B and T lymphocyte development and activation. We also discuss functional interconnections between architectural, epigenetic and transcriptional changes and introduce major key players of genomic organization in B/T lymphocytes. Finally, we present how alterations in architectural factors and/or 3D genome organization are linked to dysregulation of the lymphopoietic transcriptional program and ultimately to hematological malignancies.

## Introduction

**Figure 1:**
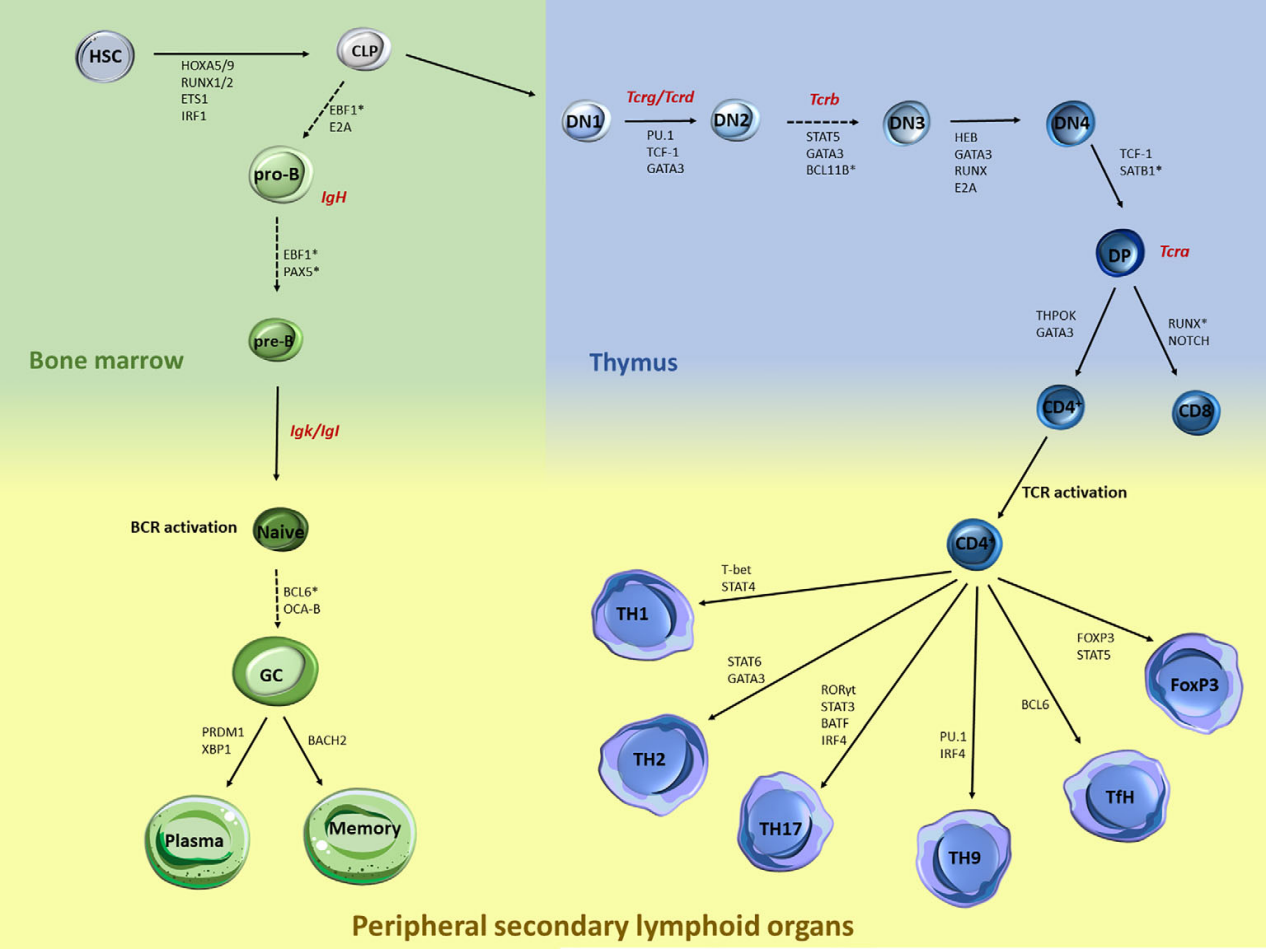
Major stages during B and T lymphocyte specification and differentiation and key transcription factors that control each transition. B and T cell development and differentiation is a stepwise process that involves multiple specification steps, cell fate bifurcations and cell migration. Multiple TFs, including but not limited to those acting at the chromatin level, have been extensively documented in HSC. Within the bone marrow, the transition of CLP towards pro-B cells is mediated through EBF1 and E2A, while the further maturation of the pre-B cells is under the control of EBF1 and PAX5. Upon BCR activation, BCL6 and OCA-B are controlling the differentiation of naive to GC cells, while the latest developmental stages are mediated through BACH2 for memory B cells and PRDM1 and XBP1 for plasma cells. Similarly, T cell development is also regulated by a strong network of TF, cytokines and genome organizers that control chromatin dynamics and T cell lineage specification. Upon thymocyte migration, the early steps of the thymocyte development are mediated through the genome organizers SATB1 and BCL11B while TF such as PU.1, TCF1 and GATA3 mediate the generation of the DN1 cells. The transition from the DN2 to DN3 is mediated by the STAT5, GATA3 and BCL11B, while RUNX, GATA3, HEB and E2A mediate the DN3 and DN4 transition. TCF-1 is also the major TF driving the DN4 to DP transition. The last steps of the intrathymic development are mediated through the expression of ThPOK and GATA3 for the CD4^+^ cells and NOTCH and RUNX for the CD8^+^ cells. Upon TCR activation, expression of the master regulator T-bet *via* STAT4 signaling leads to TH1 differentiation, while STAT6 and GATA3 regulate the TH2 differentiation. Activation of STAT3 and RORγ leads to TH17 cells, while IRF4 and PU-1 induce the differentiation towards TH9 cells. Activation of Bcl-6 induces the differentiation of naive CD4^+^ T cells into TfH. Differentiation of the Tregs is controlled by the transcription factor Foxp3 and STAT5. Generation of Tcr/Ig receptor diversity through VDJ recombination takes place at various stages during B/T lymphocyte development as depicted (red).

Over the past decades, plethora of studies have documented the transcriptional network that controls immune cell regulation and plasticity during lymphocyte development and differentiation (1–3). The lineage commitment of early hematopoietic progenitors to a specific B or T lymphocyte is a multi-step process controlled by critical cytokines and transcription factors (TF) (**Figure 1**), which ultimately promote activation of B/T cell fate programs, while posing strict checkpoints to prevent differentiation to competing lineages (4–6). Briefly, hematopoietic stem cells (HSC) within the bone marrow differentiate and give rise to a common myeloid or lymphoid progenitor (CMP and CLP, respectively). B cell development continues within the bone marrow and leads to progressive differentiation to pro-B, pre-B, and naive B cells. Upon antigen encounter in the periphery, naive B cells become germinal center (GC) B cells and can either differentiate into long-lived antibody secreting plasma cells or memory B cells (7). On the other hand, T cell development occurs in the thymus, where cells transition from several double negative (DN) stages into the double positive (DP) state when both functional T cell receptors (TCR) CD4 and CD8 are co-expressed. Eventually, only one type of receptor dominates giving rise to either CD4^+^ or CD8^+^ T cells. The thymic exodus of the naive CD4^+^ cells to the secondary lymphoid organs leads to further differentiation into several T helper (TH) cell subpopulations (3, 8).

**Figure 2:**
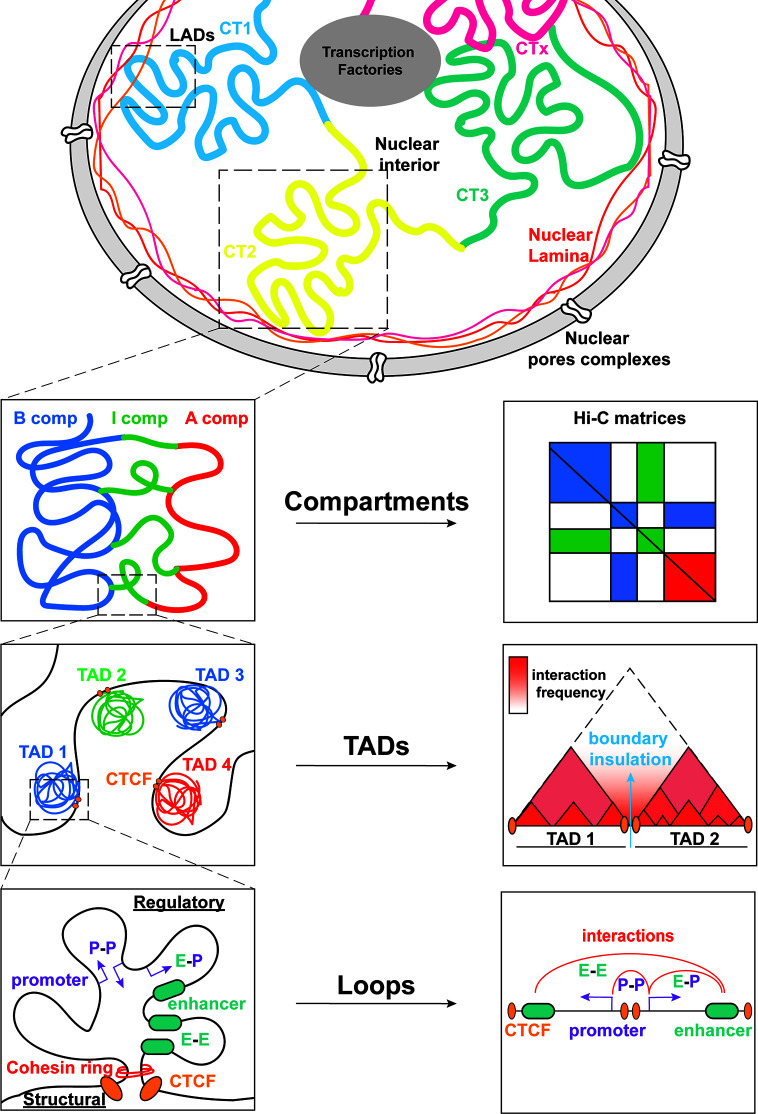
Global genome organization in mammalian nuclei from the megabase scale to the E-P level. Mammalian nuclei are organized into chromosomes with non-random distribution in the nucleoplasm. Each chromosome is further composed of chromosome territories (CT) further subdivided into A/B/I compartments. Within these compartments, TADs allow for interactions between regulatory elements (RE) that modulate gene expression. The *cis*/*trans* interactions take place between promoters (P-P), enhancers (E-E) or both (E-P).

Transcriptional regulation during lymphopoiesis relies on the activity of cell state specific TFs which can function as pioneer factors and enable chromatin landscape remodeling through the recruitment of coactivators or corepressors (1, 9, 10). Along with changes in DNA methylation and histone post-transcriptional modifications (PTM) during B/T cell differentiation, recent studies started appreciating the dynamic 3D chromatin reorganization and its association with transcriptional regulation and cell fate control in the immune system (11, 12). 3D chromatin folding and nuclear architecture play important roles in various cellular functions including gene expression, DNA replication, recombination and immune response modulation (11, 13–18). The development of chromosome conformation capture (3C) and high-resolution imaging and their derivatives (19–22) enabled the investigation of different hierarchical layers of chromatin organization based on the genome-wide identification of chromatin contacts. At the highest level of chromatin folding, individual interphase chromosomes occupy distinct regions in the nucleoplasm, called Chromosome Territories (CTs) in a non-random manner, as observed by microscopy-based methods (**Figure 2**) (23). Each of the chromosome territories (CTs) is further organized into megabase (Mb) level, through the segregation into A and B compartments, which are associated with euchromatin and heterochromatin, respectively (24, 25). Open, gene-rich and transcriptionally active chromatin regions are located within A compartments, which usually occupy the nuclear interior. B compartments are gene-poor, inactive and largely overlapping with lamina associated domains (LADs) (26), known as heterochromatic domains, located in the nuclear periphery and linked to gene repression (27, 28). Except from the A/B compartments, recently the intermediate (I) compartments were also introduced as highly dynamic chromatin domains enriched in genes poised or repressed by the Polycomb Repressive Complex (PRC) (29). At a sub-megabase level of chromatin organization, we observe self-interacting domains named topologically associating domains (TADs) (30, 31), which appear to be highly conserved across cell type and mammalian species. TADs (32) are demarcated by boundaries enriched in CTCF/Cohesin that insulate them from neighboring domains and facilitate the creation of regulatory loops (30, 31). Finally, at the finest scale of organization, chromatin is organized into looped structures or chromatin contacts that enable physical proximity among distal regulatory elements (RE), such as enhancers and promoters. These long-range interactions have been shown to play important roles in key biological processes, including DNA recombination and regulation of gene expression and cell fate (33–36).

Over the last years, a large number of studies started mapping the hierarchical levels of 3D chromatin architecture in various stages of lymphopoiesis and immune response and reveal important insights for its role in VDJ recombination, gene expression and cell fate decisions. In this review, we will discuss key principles of chromatin reorganization during various stages of B and T lineage specification, lymphocyte differentiation as well as the coordination with gene expression and cell fate decisions. We will also speculate on specific mechanisms and factors that drive architectural rewiring in lymphocytes. Finally, we will address how the 3D chromatin dysregulation might contribute to inefficient or altered immune responses, leading eventually to leukemogenesis and lymphomagenesis.

## CHAPTER I: Chromatin Reorganization During CLP Specification From HSPC

The degree to which chromatin accessibility and topology are remodeled during the step-wise differentiation from hematopoietic stem and progenitor cells (HSPC) to CMP and CLP (**Figure 1**) became recently appreciated thanks to the development of single cell (or low yield) technologies, such as scDNase-seq (37), multiple-enzyme Hi-C (3eHi-C) (38) or low input tagmentation-based Hi-C (tagHi-C) (39–41). These studies reported only limited changes at the early stages of hematopoiesis, while broad chromatin reorganization occurred at the CLP stage coinciding with a major change in cell proliferation potential (41). High resolution genome-wide contact heatmaps demonstrated that murine CLP adopt a Rabl configuration, which is defined by centromeres and telomeres localized at different poles of the nucleus (42). Trans-centromeres or trans-telomeres interactions increase upon differentiation to myeloid mature populations, although it remains unclear whether this configuration persists in the lymphoid lineage. Additionally, highly expressed genes form highly interacting domains, coined as gene body associated domains (GAD), an observation that has been independently reported in multiple cell types using high resolution Hi-C or Micro-C technologies (43–45), suggesting that high local interactivity is linked to transcriptional activity.

## CHAPTER II: 3D Chromatin Reorganization During Lymphocyte Development

### Large-Scale Subnuclear Changes

As shown by 3D chromosome painting experiments in lymphocytes and other cell types, the radial position of CTs associates with their gene density, with gene-poor CTs localized toward the nuclear periphery, while gene-dense CTs occupy more central positions (46–50). Moreover, the position of gene loci relative to the inner or outer layer of their respective CT and/or relative to the nuclear periphery associate with their transcriptional activity. For example, in human lymphocytes it was shown that transcriptionally active telomeres are located in the nuclear center, while heterochromatic centromeres are found in the perinuclear domains (47). Intriguingly, many early studies reported extensive repositioning of entire chromosomes or specific gene loci during thymic development, providing strong evidence for dynamic chromatin reorganization and its association with transcriptional changes (51). Specifically, CT6 (containing *Cd4* and *Cd8* T cell lineage-specific loci) maintains its subnuclear position during murine thymocyte development between DN and DP cells, but relocates towards the center in CD4^+^ cells and towards the periphery in CD8^+^ cells. *Cd4* and *Cd8* loci loop out from the inner core of their territory (CT6) in CD4^+^ and CD8^+^ cells, respectively, whereas the non-expressing genes are embedded within interior CT domains (51). Additional examples for gene repositioning out of the CT core during T cell activation have been reported for the *Ifng* and the major histocompatibility locus (MHC) (48, 52–54). Finally, studies in T cells have also documented gene repositioning relative to repressive or activating subnuclear domains along with transcriptional changes. Specifically, repression of either *Cd4* or *Cd8* gene in mature CD8^+^ and CD4^+^ cells, respectively, was linked to their repositioning towards pericentromeric heterochromatinic (PCH) regions (55, 56). Similarly, during B-cell maturation, genes have also been observed to reshuffle towards or away from heterochromatic foci enriched for the repressive protein IKAROS, in order to be inactivated (e.g. *λ5*) or expressed (e.g. *Cd2*) in a stage-specific manner (57).

### Large-Scale Compartmentalization Changes

**Figure 3:**
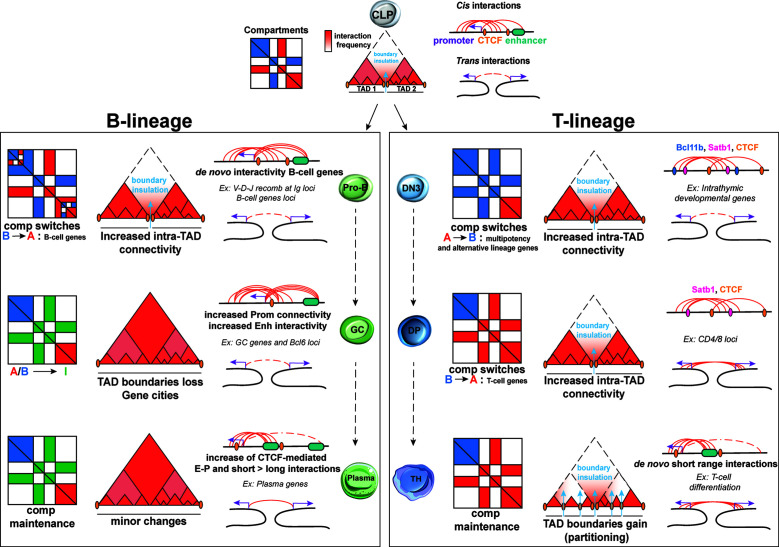
Dynamic 3D chromatin changes during lymphocyte development and differentiation. B lineage: Upon commitment to the B-cell lineage, minor changes are documented for A/B compartments and TADs at the pro-B stage. Nonetheless, major changes in intra-TAD activity are associated with B-cell specific genes. Upon activation, GC B-cells are uniquely characterized by an increase in the weak Intermediate (I) compartments, which are maintained during plasma cells transition. TAD boundaries tend to be lost in GC B cells, leading to “gene cities” specific organization. While E-P loops tends to be increased in both GC and plasma cells, the latter subpopulation is characterized by a shift from long to short-range interactions. T lineage: During T lymphocyte development, the earliest global changes in A/B compartment, intra-TAD connectivity and loop formation take place at the T cell commitment step upon the transition of DN2 to DN3 cells but also in the DN4 to SP transition. Conversely, TCR activation does not cause changes in A/B compartments but results in TAD partitioning due to *de novo* and stronger TAD boundaries. A high proportion of both intra- and interchromosomal interactions are found for these 3 stages of T-cells development and differentiation for which the high E-P loop formation ensures T helper lineage specific gene expression.

The development of 3C technologies enabled the tracking of topological reorganization at various developmental stages and at a genome-wide level (24, 30, 58, 59). During B cell development, compartmentalization remains largely unchanged with only few A and B compartment switches between murine HSC and pro-B cells (0.7%) (60). Focusing more specifically into the transition from pre-pro-B to pro-B, Lin et al. detected local reorganizations, resulting in about 20% of A-to-B or B-to-A compartment switches (**Figure 3**). These changes were associated with increased or decreased nascent transcriptional activity, respectively, as measured by global run-on sequencing (GRO-seq) (61). Some of the largest domains that switched from a repressive to permissive chromatin state included critical B-cell regulatory genes such as *Ebf1*, *Foxo1*, *Igκ* and *Igλ* loci. Interestingly, most of B-to-A switched loci that showed no transcriptional upregulation were enriched instead for H3K27me3 deposition, a chromatin mark linked to poising and gene repression, indicating that the transition to a permissive compartment is not always accompanied by transcriptional changes, but also by remodeling of chromatin state.

Large-scale chromatin reorganization also occurs during T cell development as revealed by a recent study integrating scDnase-Seq, 3e Hi-C and RNA-Seq datasets from distinct stages of T cell development, starting from HSPCs and including CLP, DN1, DN2, DN3, DN4 and DP cells (39). This analysis detected changes in A/B compartmentalization with more than 1,200 genomic bins exhibiting A/B compartment switching. Intriguingly, the majority of A-to-B switches occurred during the transition from HSPCs to DN2 cells and B-to-A during the transition from DN3 until DP stage of thymic development, suggesting a global chromatin closure during early stages of T cell differentiation followed by opening upon T-cell lineage commitment (**Figure 3**). Overall, the highest percentage of global switching of A/B compartment during T cell development was observed at the DN2-to-DN3 transition, which is one of the most pivotal checkpoints for T lineage commitment. During this stage, genes that promote multipotency and differentiation of alternative lineages (such as *Hmga*, *Meis1*, *Lmo2*) undergo A-to-B compartment switching, while genes involved in T-cell maturation and selection (such as *Bcl11b, Ets1, Tcf7, Cd3d*, and *Lef1)* relocate from B-to-A compartment (39). Interestingly, integration of gene expression datasets showed that transcriptional silencing sometimes occurs prior the A-to-B compartment switching. Similarly, B-to-A compartment switches included promoters that were pre-marked by H3K4me3 in HSPCs, indicating that local permissive chromatin state can be independent from topological changes (both in compartments and TADs, as discussed later).

### TADs and Chromatin Loops

Major TAD reorganization in B cells occurs at the *Ig* loci during V(D)J recombination (62, 63), which constitute the most important step of early lymphocyte development that controls the rearrangement and the expression of a diverse repertoire of B and T receptors (B/TCR) (64, 65). This process involves the *de novo* establishment of multiple long-range contacts along the *Igh* or *Igκ* loci, which leads to locus contraction (66, 67) (see Chapter IV). Another example of crucial TAD reorganization has also been reported to occur around the genes that encode the recombination-activating genes (RAG) DNA cleaving enzymes necessary for the V(D)J recombination (68). The B or T cell specific expression of these genes depends on chromatin interaction involving lineage-specific *Rag* cis-regulatory elements (CRE) in B and T progenitors mediated by the E2A pioneer factor (69). One T cell specific (*R-Ten*) and two B cell specific (*R1B* and *R2B*) enhancers were identified that interact with *Rag* promoters, leading to cell type-specific sub-TAD organization. Either deletion of enhancers or mutations of the E2A binding motif were sufficient to disrupt TAD organization, long-range interactions and *Rag1/2* expression. Interestingly, in macrophages the *Rag1/2* locus is silenced and insulated from the neighboring active compartment through a strong TAD boundary, which is absent in pro-B/pro-T cells.

In agreement with previous studies in other cellular contexts, the number, position and average size of TADs along B cell development remains largely invariant genome-wide (30, 61, 70). Nonetheless, significant changes in intra-TAD *cis* interactions were observed upon specific transitions stages. For example, *Polg2*, which is highly expressed and interacting with multiple enhancers in pre-pro-B, lost all chromatin contacts in pro-B. Conversely, the *Cd79b* and *Ebf1* loci were shown to establish multiple *de novo* interactions in the pro-B stage (61, 70). At the *Igκ* locus, a distal enhancer *E88* forms a highly connected enhancer hub within the V region and its deletion results in major alterations of long-range interactions between V and J regions and reduced receptor variant diversity (71). Overall, these results suggest that while TAD genomic position maintenance is largely unaffected, intra-TAD interactions changes are most likely still crucial for cell type-specific gene expression, sustaining B cell identity and functionality.

Changes in intra-TAD organization have also been reported during T cell development (39), with many loci important for T cell lineage commitment gaining or losing interactions. A significant increase in intra-TAD connectivity was observed during DN2-to-DN3 and DN4-to-DP transitions, coinciding with major changes in chromatin accessibility and compartmentalization, as described above. Globally, increased or decreased intra-TAD connectivity during each of the HSC to DP transition states strongly correlated with gene upregulation or downregulation, respectively. Importantly, architectural reorganization within TADs usually preceded transcriptional activation of associated genes, suggesting that pre-establishment of chromatin contacts generates a permissive topology for gene activation (39, 72, 73).

During thymocyte development, transcriptional regulation of CD4/CD8 coreceptors is concomitantly linked with cell-fate choice toward either the helper or cytotoxic lineage (10, 74–76). Both murine *Cd4* and *Cd8* loci undergo 3D reorganization that involves stage-specific chromatin contacts between *Cd4* and *Cd8* promoters with various proximal and distal RE (77). In DN thymocytes, *Cd4* activation is prevented by a chromatin loop between an upstream *E4p* enhancer and a silencer located 3kb downstream of the TSS (78), mediated by RUNX1 and P-TEFb (79). However, in DP and CD4^+^ cells, this chromatin interaction is dissolved and new ones are established between the *Cd4* promoter and the *E4p* enhancer (in DP stage) and/or another proximal enhancer *E4m* located ~2 kb downstream of the TSS (in CD4^+^ cells) leading to the initial activation and further upregulation of *Cd4* during these transitions (80, 81). These new activating chromatin contacts are mediated by RUNX1 and other transcription factors, such as E2A and TCF-1. Dynamic chromatin interactions have been also reported around the *Cd8* locus, which is composed by the *Cd8α* and *Cd8β* genes, showing the same transcriptional orientation but separated by 36 kb. In murine CD8^+^ cells, the expression of *Cd8* locus is regulated by six different enhancers through stage-specific enhancer-promoter (E-P) interactions and mediated either by IKAROS and BAF complex in DP cells or ThPOK in CD8^+^ cells (82). These chromatin contacts are significantly lower in B cells, where *Cd8* locus is inactive (83). Further functional validation (e.g. deletion/mutation of enhancers) will help to elucidate the role of each regulatory contact in the control of transcription and thus cell fate.

As well as the intrachromosomal E-P interactions reported in *Cd4* and *Cd8* genes, long intrachromosomal interactions have also been documented between these two loci in murine DP and CD8^+^ cells, but not in DN and CD4^+^ cells. The stage-specific, interchromosomal association between these genes was also confirmed by 3D DNA FISH in humans where *Cd4* and *Cd8* genes are located on chromosomes 12 and 2 respectively (55). The conservation of the *Cd4-Cd8* interaction in murine and human T cells highlights the significance of the 3D chromatin organization in coordinating transcriptional regulation of genes, which not only defines the stochastic CD4/CD8 lineage commitment but also directly control the immune responses.

Although the majority of 3D genomic studies in T cell development were conducted in murine models, a study in human primary resting CD4^+^ cells started unraveling the principles of 3D chromatin organization around active enhancers and promoters by applying ChIA-PET technology with antibody against the active histone mark H3K4me2 (84). This study identified a total of 6520 E-P connections, the majority of which were short-range (56%) and independent of CTCF binding (81%), indicating the implication of other possible genome organizers, such as TFs (SATB1, ETS, RUNX and GATA) or transcriptional coregulators (see Chapter IV). Interestingly, extensive long-distance E-P interactions, were also reported for *Vav1* and *Runx1* genes, both regulators of thymocyte development and TCR signaling cascade. In the same study, the global mapping of the long-range interactions in chromosome 19 (known to contain a rich network of multiple enhancer and promoters) showed five distinct chromatin domains enriched with “local” interactions from 20 kb to hundreds of kb, while these domains were interconnected by super long-distance interactions, indicating multiple and complex layers of 3D chromatin organization in human CD4^+^ cells (84).

In conclusion, major architectural reorganization occurs during various steps of B and T cell development, involving A/B compartment switches, changes in intra-TAD interactions and dynamic establishment or loss of chromatin contacts *in cis* and *in trans*. Although many of these topological changes associate with transcriptional activation or silencing, functional perturbations will be critical for dissecting the temporal interconnections and cause-and-effect relationships between the two processes. Notably, the various transitions during the step-wise B or T cell development are characterized by different degrees of global chromatin reorganization, with the most striking changes coinciding with key checkpoint transitions, such as DN2-DN3 (39, 85). Whether this major topological rewiring is critical for irreversible “locking” of cell identity or solely a consequence of other major molecular and cellular changes, such as global chromatin opening and increased proliferation capacity, remains to be shown.

## CHAPTER III: Chromatin Reorganization Upon B/TCR Activation

One of the most interesting key features of lymphopoiesis emerges from the multitude of transitions between resting and proliferative phases during which lymphocytes modulate their global transcription levels, metabolic activities and cytokine production (86–90). These changes are also accompanied by dramatic remodeling of chromatin accessibility and 3D architecture along with a change of nuclear volume. Before antigen encounter, both naive B and T cells constitute quiescent populations endowed with a very dense and compacted chromatin accompanied by long-range, intrachromosomal interactions (84, 91). Of note, in T cells this highly compacted chromatin state, which is mediated by the condensin II complex, is also critical to prevent premature or aberrant signal activation by blocking Stat5 access to its genomic targets (92). TCR activation leads to a rapid and dramatic enlargement of the nuclei from 4-5μm diameter in the murine naive CD4^+^ to 10-12 μm diameter in TH1 and TH2 cells, as shown by confocal and transmission electron microscopy experiments *in vitro* (92–94). A similar massive nuclear expansion accompanied by Myc- and ATP-dependent chromatin decondensation is observed upon BCR activation of naive B cells by IL4 and LPS, as observed by super resolution microscopy (95). Additionally, ^14^C incorporation and micrococcal nuclease (MNase) experiments demonstrated a concomitant global increase of histone acetylation, chromatin accessibility and transcriptional activity (88, 95) as well as a significant and gradual DNA hypomethylation when profiling activated B cells for whole genome bisulfite sequencing (WGBS) (96).

### B Cells

GC B cells: Hi-C analysis on human B cells, revealed a multilayer 3D chromatin reorganization upon the transition from naive to GC B cells including: an increased promoter connectivity and enhancer interactivity, 5’ to 3’ GC gene looping and merging of gene neighborhoods marked by active histone modifications (97). Genomic regions with increased interactivity were associated with dense binding of SPIB, PU.1 and EP300, the role of which in 3D chromatin organization remains to be elucidated. Loss of TAD boundaries were more frequent than gains and led to the transformation of “Gene neighborhoods” (30, 98) into larger and *de novo* “Gene cities” (97).

One of the most striking examples of chromatin remodeling and 3D rewiring during the GC transition occurs around the *Bcl6* locus, which encodes for a master regulator of GC reaction. A 114 kb long, distal *Bcl6* enhancer cluster, which functions as a Locus Control Region (LCR), undergoes dramatic epigenetic alternations, including chromatin opening, binding of critical TFs and cofactors [such as OCT2 and OCA-B (99)], gain of H3K27ac and increase in transcriptional activity. In parallel, the LCR establishes a large number of *de novo* and strong interactions with *Bcl6* promoter and other nearby genes forming a highly interacting hub. More specifically, OCA-B was shown to directly recruits Mediators, bridging the *BCL6* LCR to the *BCL6* promoter *in cis* (99). Although the exact organizational principles and regulatory properties of this hub remain to be fully elucidated, deletion of the *Bcl6* LCR in mice resulted in the abrogation of the GC formation beyond the regulation of Bcl6 *per se* (97).

A recent study demonstrated that in B cells, the naive to GC transition is also characterized by compartmentalization changes and the emergence of a new type of weak, Intermediate (I) compartments, which allow for inter-compartment interactions. I compartments in GC B cells arise from A or B compartments and are enriched for the H3K27me3 mark, containing genes repressed by the PRC (29).

Plasma cells: The GC transition to plasmablasts (and further to long-lived plasma cells) is characterized by a major chromatin reorganization harboring a typical cartwheel-like structure as defined by Cajal and Marschalko (100). The silencing of *Pax5* expression at this stage was proposed to be responsible for overwriting the typical B cell transcriptional program and 3D chromatin organization (60), although only minimal compartment changes were identified. Indeed, plasma cells have been shown to retain a high fraction of I compartments (29), irreversibly locking the 3D chromatin organization.

A number of recent studies have started to shed light into the extent and principles of 3D chromatin reorganization upon naive B cell activation *in vitro*, using various stimuli and timepoints after activation. Activation of murine naive B cells with IL-4 and LPS for 24h led to very few changes in compartments and boundaries, but showed a significant shift from long-range to short-range interactions as demonstrated by *in situ* Hi-C and ChIA-PET experiments (91). There was also a substantial increase in the number and strength of CTCF-mediated E-P loops and increased binding of cohesin at loop anchors (95). Similarly, a recent study using a genome-wide tethered chromosomal conformation capture analysis (TCC) on murine LPS-activated plasmablasts (101), identified a higher number of short intrachromosomal interactions (<10 Mb) in comparison to naive B cells. Moreover, this study also described a number of interchromosomal interactions that were preferentially enriched for genes associated with the plasma cell fate such as *Prdm1* and *Xbp1*.

More recently, a study tracked the dynamic transcriptional and architectural changes from the very early time points after LPS activation of murine naive B cells until their differentiation into antibody-secreting plasma cells (102). The authors reported that early transcriptional changes (3hr post-activation) precede genomic reorganization, suggesting either the pre-existence of a permissive configuration with an instructive role of transcription in 3D chromatin reorganization or a partial uncoupling of the two processes. *In situ* Hi-C analysis uncovered two major waves of 3D chromatin changes: before the first cell division (at late G1 phase) and upon plasmablast differentiation. As previously reported, no major chromatin changes were uncovered at the compartment nor at the TAD levels. Most changes involved stage-specific loss or gain of long-range E-P interactions (<1 Mb) around gene loci relevant for each transition, including *Bcl6*, *Ell2* and *Pax5*. Intriguingly, conformational changes that occurred in the first wave were largely preserved during clonal expansion despite multiple rounds of mitosis, during which chromatin architecture (including compartments, TADs and loops) is lost (103, 104). This suggests that the B cell transcriptional program and topology are faithfully reestablished during each division, although the underlying mechanisms remains unclear. The second wave of genome reorganization occurs during a prolonged G1 phase linked to plasma cell differentiation, highlighting the importance of this time window both for architectural changes and cell fate decisions, as reported in other systems (104–106).

Memory B cells: The dynamic changes in chromatin state and conformation landscape that occurs upon B cell activation and GC transition are largely reversible as cells progress to the memory stage. The epigenetic landscape, transcriptome, chromatin accessibility and 3D organization of quiescent human naive and memory B cell populations are indeed indistinguishable upon unsupervised clustering, nonetheless clearly separated from GC and plasma mature B cells populations (29). Most of I compartments defined in GC B cells, switch back upon memory B cell transition to a compartment organization similar to naive B cells, erasing this layer of 3D organization imprint and allowing memory B-cells priming for GC re-entry. In contrast to all of these multi-omics characterizations, DNA methylation and H3K27me3 profiles clearly separate memory from naive B cells, indicating that the epigenetic state of memory B cells is only partially reversed to a naive-like configuration. Together, these epigenetic and topological features might be critical for the ability of memory B cells to rapidly re-enter GC reaction and differentiate into plasma cells upon secondary infection (107, 108).

### T Cells

Multiple studies using 3D DNA-FISH and 3C assays described various degrees of topological reorganization during T cell activation and differentiation towards T helper lineages (TH1 or TH2). For example, specific genes encoding important effectors and cytokines of the opposite lineage (e.g. *c-Maf* and *Il-4* in TH1 cells and *Ifng* in TH2 cells) have been reported to relocate towards heterochromatic compartments (109, 110). This subnuclear repositioning is linked to gene silencing, supporting TH polarization and sub-lineage specification. Architectural changes linked to upregulation of gene expression upon TCR activation were also described early on. 3C experiments showed that binding of transcription factors STAT3 and NFATc2 factors mediate E-P looping around the *Il-21* locus (111), promoting the transcriptional activation of this significant pleiotropic cytokine that acts as a regulator of inflammation and immune responses. Additionally, an extremely long-range intrachromosomal interaction (~98.5 Mb) that brings the promoter of *IfngR1* gene proximal to *Ifng* (master regulator of the TH1 differentiation) promoter and its downstream RE was observed in murine CD4^+^ and TH1 cells. This interaction occurred in a monoallelic fashion in agreement with the monoallelic expression of these genes, while it was absent in TH2 cells, where none of these genes are expressed (112). Finally, an example of interchromosomal association has been documented in murine CD4^+^ cells between the *Th2* locus that contains interleukin 4 (*Il4*), interleukin 5 (*Il5*) and interleukin 13 (*Il13*) genes located in a single gene cluster on chromosome 11 and the TH1 cytokine interferon gamma (INF-γ) located on chromosome 10. This physical interaction between these loci presents a “poised” chromatin conformation and precedes the T cell effector fate decisions. Upon TCR activation, select cytokine genes dissociate from this hub in order to be expressed, enabling polarization either towards the TH1 or the TH2 fate (72). In the past years, several studies started revisiting the 3D chromatin reorganization upon T cell activation using technologies that allow quantitation on a genome-wide scale. A recent study in human CD4^+^ reported that upon 24h of TCR activation, about 30% of long-range chromatin interactions undergo significant changes, while compartments or TADs remain unaffected (113). In agreement, another study combining ATAC-seq, *in situ* Hi-C and RNA-seq in human CD4^+^ and CD8^+^ cells before and after *in vitro* TCR stimulation for 72h, reported only minimal changes in compartmentalization. Specifically, less than 4% of the genome switched from A-to-B compartment along with their respective transcriptional changes (114). However, the same study reported significant differences at the sub-megabase level with the emergence of new and stronger boundaries that partitioned TADs into smaller and more numerous subdomains when compared to naive T cells. Moreover, TCR activation led to formation of *de novo* short-range chromatin loops, resulting in increased intra-TAD connectivity in more than 60% of TADs in both CD4^+^ and CD8^+^ cells. The establishment of new chromatin contacts coincides with increased chromatin accessibility around regions that contain binding motifs for transcription factors related to T cell development and differentiation (114, 115). On the other hand, the observed strengthening of TAD borders was found to be coupled with reduced chromatin accessibility, higher nucleosome occupancy and low levels of gene expression. Importantly, this topological reorganization and transcriptional reprogramming upon TCR activation was largely restricted to genes relevant for immunity. The dynamic changes in the chromatin interactions upon TCR activation were also recently reported by several other groups, based on promoter capture Hi-C (PCHi-C) and Trac-looping experiments, demonstrating that these promoter interactomes were lineage-specific and associated with target gene expression (116–118). All studies indicated rewiring on E-P chromatin interactions, global changes in chromatin accessibility and histone modifications (H3K27ac, H3K4me1, H3K4me3), when compared to naive T cells.

So far, only few studies on chromatin organization in T helper cells have been reported. In 2017, two different reports described the genome organization in murine TH2 and human TH17 cells (38, 119). 3eHi-C experiments in murine TH2 cells identified a total number of 1,363 TADs and CTCF binding sites were found not only at the TAD borders but also interspersed on enhancers within TADs. Knock-down of CTCF expression led to significant downregulation and cell-to-cell variation of immune related genes, while deletion of specific intra-TADs CTCF binding sites led to compromised E-P interactions (38). These experiments support an important role of CTCF in stabilizing E-P contacts and controlling cell-to-cell fluctuations of gene expression. The same study reported that within TADs, long range E-P interactions mostly involved genes critical for immune function and T cell activation, suggesting that transcription of lineage-specific genes depends on physical communication with distal enhancers, while housekeeping genes mostly rely on proximal regulatory elements (38).

Comparison between four different subsets of T cells (bulk CD4^+^, naive CD4^+^, TH17 and Tregs using either PCHi-C or H3K27ac HiChIP) revealed cell type-specific loops upon differentiation that might contribute to the unique identities and functions of T cell subsets (119). Differentiation of naive CD4^+^ cells into TH17 cells or regulatory T cells (T_regs_) creates subtype-specific E-P interactions between regions with similar DNA accessibility as shown by H3K27ac HiChIP on primary human T cells. Genes within cell-type-specific E-P loops encode for canonical T cell subtype TF and effector molecules, further supporting the potential regulatory impact and biological significance of these interactions. Moreover, the anchors of cell-type specific H3K27ac HiChIP loops enriched for binding of TF known to drive T cell subtype differentiation, suggesting a role of these TFs on the loop formation (119).

In conclusion, all abovementioned studies started unraveling the principles of 3D reorganization during both T and B cell lineage commitment and differentiation. In both lineages, major structural changes in A/B compartments are observed, while TAD size and numbers are largely conserved throughout the process with the exception of B/TCR activation, which leads to extensive TAD boundary alterations. Loss of boundaries causes TAD fusion in GC cells whereas the acquisition of *de novo* and stronger boundaries led to TAD partitioning in activated T cells. Moreover, B and T cell activation is accompanied by an increase of intra-TAD connectivity especially around genes relevant for lymphoid identity and function. During B/T lymphocyte development and differentiation, studies reported increased long and short *cis* chromosomal interactions, while during T cell development and TH polarization inter-chromosomal interactions have also been observed and linked to transcriptional changes around critical T cell regulatory genes. These *trans* interactions, but also a large percentage of *cis* E-P loops in T cell lineage are independent of CTCF binding, highlighting the need for investigating other nuclear proteins and mechanisms for their roles in shaping 3D genome folding in lymphocytes.

## CHAPTER IV: Presumed Mechanisms and Players of 3D Chromatin Reorganization During B/T Lymphopoiesis

In the previous chapters, we discussed the global and local 3D chromatin conformation changes that occurs during lymphocyte specification as well as activation and differentiation. *What are the forces and critical players that drive this topological rewiring?*

There are several well-described mechanisms and architectural factors that actively drive different layers of 3D genomic organization, as extensively reviewed in multiple recent papers (120–124). In mammalian cells, CTCF/Cohesin-mediated loop extrusion (121, 125) is shown to be responsible for the formation of most chromatin loops and TADs/subTADs, since degradation of any of these architectural components causes drastic changes in chromatin topology and variable -rather moderate- effects on gene expression (126–130). On the other hand, increased “self-attraction”/affinity among chromatin loci with similar transcriptional and chromatin states, such as homotypic histone modifications and TF/cofactors is important for compartmental segregation and the emergence of droplet-like, phase-separated, membraneless organelles, such as nucleoli or nuclear speckles (131–134).

A large number of cell-type specific TFs (135, 136) and transcriptional-cofactors (e.g. BRD4, PRC2, Pol II) (134, 137–139) have been reported to form large nuclear condensates, which may mediate activating or repressive chromatin contacts and hubs in a loop-extrusion independent manner. Moreover, TFs have been reported to mediate long-range chromatin contacts through direct homodimerization (e.g. YY1) or protein-protein interactions with other *bona fide* architectural factors, such as cohesin or PRC2 components (120, 140–143). Importantly, the expression and/or genomic distribution of many of these critical factors (TFs and histone modifications) change during B and T cell development and activation, therefore they might contribute to the observed 3D chromatin reorganization.

Below, we will discuss key findings that support the roles of CTCF/cohesin and select TFs in stage-specific 3D architecture during lymphopoiesis. Given that these mechanisms have been recently reviewed elsewhere (VDJ and TF reviews) (144–146), we will focus on two less appreciated proteins and structures (histone linker and nuclear lamina) that play important roles in the regulation of 3D nuclear organization of B and T cells.

### CTCF/Cohesin-Mediated Loop Extrusion

The loop-extrusion mechanism describes the progression of chromatin through the cohesin ring and the stabilization of loops when the ring encounters convergent CTCF sites. In the context of lymphocyte biology, CTCF/cohesin complex was reported to mediate long-range chromatin interactions that control stage-specific transcription of various cytokine and other critical genes (*Ifng*, *Il21*, *MHC-II* etc.) during B/T lymphocyte development and differentiation (111, 147–152). One of the most striking and well-studied examples of chromatin looping through CTCF/cohesin extrusion is the V(D)J recombination in developing B cells (66, 153–156). This ensures recombination of any of the 113 VH segments with any of the 13 DH and 4 JH segments and thus, generation of an extremely diverse repertoire of antigen receptors. This process, which has been extensively reviewed recently (157, 158), occurs through a number of highly regulated steps and loops, which are dependent on cohesin factors and multiple CTCF binding sites (159, 160) resulting in a remarkable contraction of the entire (~2.75 Mb) *Igh* locus (161). Similar CTCF and cohesin-dependent loop extrusion mechanisms mediate V(D)J recombination at the *Tcr* loci in T cells. The creation of a developmentally regulated chromatin hub supports V_α_–J_α_ synapsis and eventually leads to a successful V(D)J recombination (156, 162, 163). Deletion of a specific CTCF binding site (named EACBE), causes merging of the subTAD domains, reduction of long-range chromosomal interactions between the V_α_ and J_α_ segments and leads to impaired *Tcra* rearrangement (164).

Genetic knock-out or protein degradation experiments in cell lines and mouse models further support the critical roles of CTCF and Cohesin in lymphocyte development. Specifically, it was recently shown that CTCF is required for proper control of the transcriptional program and high proliferation rate of GC B cells and its depletion results in premature differentiation into plasma cells (165). On the other hand, genetic ablation of *Ctcf* in mouse thymocytes leads to cell cycle arrest, while deletion of CTCF binding sites results in increased cell-to-cell variation of gene expression, indicating a fundamental significance of these long-range E-P interactions in stabilizing gene expression in mammalian T cells (38, 166). CTCF/cohesin complex binding is also highly enriched at enhancers and in particular “super-enhancers” in murine thymocytes, mediating long-range communication with target genes (167). In the same study, it was also reported that cohesin facilitates enhancer clustering in thymocytes, while its conditional deletion results in weakened enhancer-enhancer interactions and downregulation of the associated genes.

Interestingly, transcription and 3D chromatin organization can also be maintained in a cohesin-independent manner. Indeed, using PCHi-C in a CTCF or Cohesin auxin-inducible degron (AID) system to assess the rewiring of promoter-anchored loops, Thiecke et al. showed that while a majority of promoter contacts are lost, a significant number of them are actually constrained and maintained by TAD boundaries or even gained. Cohesin-independent interactions were mainly centered on active promoters or active promoters and enhancers but remained affected by transcription modulation (168). These results mirror the minimal impact of cohesin depletion on enhancer activity and transcriptional control in steady state conditions (126–129). Nonetheless, cohesins were shown to be crucial for macrophage-induced inflammatory response of HSPC (169) arguing for a role of these genome organizer in cell fate transition. While genetic and epigenetic mechanisms enabling cohesin-independent looping are not completely understood, the role of several chromatin co-factors involved in phase condensates such as mediators and Brd4 has been recently addressed. Using a SMASh tag degron system, El Khattabi et al. were able to discriminate essential from non-essential mediators in T and B lymphocytes based on their capacity to affecting Pol ll recruitment to the chromatin (170). *In situ* Hi-C and ChIA-PET profiling MED14-depleted B-cells demonstrated that Mediator complex is largely dispensable for E-P looping. Similarly, E-P loops are not drastically affected by the inhibition/degradation of the chromatin insulator Brd4 nor by phase condensate dissolution in a B-ALL cell line (73), conversely to widespread transcription and chromatin decompaction.

### Lineage-Specific Transcription Factors

Lymphopoiesis is controlled by a number of lineage-specific TFs, which orchestrate stage-specific transcriptional and chromatin alterations, including changes in chromatin accessibility, DNA methylation, histone modifications, and ultimately 3D chromatin organization. Critical lineage regulators, such as Ebf1 in B cells (171, 172) and Tcf1 in T cells (173), have been reported to act as pioneer factors capable of binding previously inaccessible genomic regions and inducing local chromatin opening. According to these studies, recruitment of epigenetic modulators and cofactors will then lead to additional epigenetic remodeling including erasure of repressive marks (e.g. H3K27me3 and DNA methylation) and deposition of active histone modification, which coincides with activation of lineage-specific genes. In parallel with the TF-mediated changes in chromatin accessibility, state and activity, a lot of recent studies support functional links between TF binding and 3D chromatin reorganization during cell fate transitions, such as reprogramming (45, 174), differentiation (175, 176), or tumorigenesis (177) and started shedding light into the temporal dynamics and underlying mechanisms. Protein oligomerization and interactions with loop extruders or nuclear landmarks (nuclear lamina) are some of the proposed mechanisms by which TFs might mediate chromatin looping, such as E-P interactions, as shown for example for YY1, KLF4 (45, 141, 178, 179). Alternatively, TFs might promote long-range contacts by biomolecular condensation through low-affinity multivalent interactions non-coding RNAs and other protein factors (e.g. BRD4, p300 and Mediator complex) *via* their intrinsically disordered regions (IDRs) (120, 180). Finally, TF binding might indirectly contribute to 3D reorganization by inducing changes in chromatin states and thus altering compartmental segregation.

A large number of key lineage TF and protein cofactors in B/T lymphocyte development have been proposed to mediate chromatin reorganization and DNA looping as recently reviewed (77, 145, 181–183), although functional validations and mechanistic insights of their proposed architectural function are often missing. Below, we discuss some of the best-studied examples:

Special AT-rich binding protein 1 (SATB1) is a T cell enriched transcription/epigenetic factor critical for thymocyte development and differentiation. Multiple studies support the role of SATB1 also as 3D genome organizer by mediating specific chromatin loops, contributing to a complex protein scaffold that forms an “aromatic” or “cage like” structure, known as “loopscape”. The loopscape circumscribes heterochromatin away from euchromatin by tethering distant genomic loci into its network, facilitating their coordinated transcriptional regulation (184–187). The most well documented loopscape example has been reported for the *MHC* locus, where SATB1 interacts with promyelocytic leukemia (PML) protein in order to organize the locus into distinct higher-order chromatin-loop domain structures. Silencing of either SATB1 or PML leads to dynamic reorganization of chromatin loops affecting the expression of the MHC class I genes (54). SATB1 was also shown to play a critical role in the control of *Tcra* locus rearrangements through RAG regulation (188). In DP thymocytes, the highly expressed SATB1 binds to the anti-silencer element (*Ase*) and to the distant *Rag1* and *Rag2* promoters, mediating the formation of a chromatin hub. The latter facilitates RNA polymerase II recruitment and leads to activation of *Rag* genes. Genetic depletion of Satb1 leads to a reduction of RNA Pol II recruitment and transcriptional activation, eventually causing a defective V(D)J recombination during thymocyte development. Additionally, in TH2 differentiated cells, SATB1 has been shown to function as anchor for the formation of dense small loops around the *Th2* gene locus leading to the activation of all cytokine genes (*Il4, Il5* and *Il13)* that are harbored in the locus (185).

BCL11B is a zinc finger TF that function as a key developmental regulator of cellular differentiation in the T-cell lineage (39, 189–191). BCL11B is considered as a T cell-specific genome organizer that maintains the T cell nucleome and mediates the formation of DNA loops during thymic T cell development. BCL11B is highly expressed upon DN2 to DN3 transition leading to *de novo* genomic E-P interactions. Deletion of *Bcl11b* in CD4^+^ cells led to a decrease in E-P interactions suggesting that even in late T-cell developmental stages the topologically interacting DNA loops are BCL11B-mediated (39). Although most of the studies have shown the role of BCL11B during T cell development, few groups have also documented its role in later stages of T cell differentiation. BCL11B binds to RE of critical genes such as *Il-4* (silencer), *Runx3* (enhancer) and *Gata3* (promoter) during TH2 cells differentiation. This binding facilitates and stabilizes the TH2 lineage fidelity indicating structural roles of this factor in the regulatory loops/network upon T cell activation (192).

Yin Yang 1 (YY1) is known as a transcriptional activator or repressor and contributes to chromosome organization through mediating interactions between active E-P loops in several cell types (141, 193). Genetic ablation of its binding sites or depletion of YY1 protein leads to disrupted loops and gene expression. YY1 has a pivotal role during B cell development and lymphoma (194), with the most well characterized chromatin loop example reported for the *Igh* locus (195). 3D DNA FISH and 3C experiments showed that YY1 controls the Ig class switch recombination, *via* bridging the *Igh* intron with the 3′ regulatory region (3′RR) located at the end of the *Igh* locus. Additionally, YY1-mediated loops have also been reported for *Th2* locus during TH2 differentiation, where YY1 knock down TH2 cells presented decreased intrachromosomal interactions as shown by 3C experiments (196).

Paired Box 5 (PAX5) is a TF that has a dual role in activating the B cell program while preventing expression of non-B cell genes (197). The role of Pax5 in maintaining global 3D genome organization in B cells was recently investigated by a study that combined Hi-C and Pax5 ChIP-seq profile of WT and Pax5^-/-^ Pro-B (60). While no major changes in compartmentalization nor TAD numbers were uncovered in Pax5^-/-^ pro-B cells, Pax5 loss associated with local chromatin reorganization (strengthening or weakening of loops) at specific loci, including the *Igh* locus, where Pax5 binds at distal V_H_ regions. This is in agreement with a previous study, which documented that Pax5 deletion in pro-B leads to preferential usage of the 4 V_H_ segments, loss of long-range interactions within the *Ig* locus TADs and overall abrogation of *Igh* locus organization (198). Intriguingly, re-expression of *Pax5* in Pax5^-/-^ pro-B showed a partial rescue of the observed topological changes, indicating that this TF is necessary but not sufficient to establish and maintain the genome organization in developing B cells. In further support to this notion, *Pax5* ectopic expression in T-cells, was insufficient to induce *Igh* locus contraction. Intriguingly, restoration of chromatin interactions in *Pax5*-rescued pro-B was not impaired upon treatment with the RNA Pol ll inhibitor α-amanitin, suggesting that the role of Pax5 in 3D organization is largely independent of its function as transcriptional regulator (198).

### Linker Histones

Dynamic changes in chromatin state may also induce 3D architectural changes either through the differential affinity between homotypic or heterotypic histone modifications or through protein-protein interactions among the recruited epigenetic readers (121, 123, 129, 199, 200). Although, literature has mainly focused on core histones and their modifications, two recent studies started shedding light into the role of H1 linker histones in local and global chromatin architecture, in the context of B and T cell biology (201, 202). Using a triple conditional knock out (cTKO) for Histone 1 isoforms c/d/e, Willcockson *et al.* specifically depleted H1 in murine T-cells and uncovered a de-repression of T-cell activation genes along with chromatin decompaction, reminiscent of T-cell activation. Hi-C analysis of CD8^+^ cells indicated that Histone 1 binding drives chromatin compaction not only within B compartments, but also within a subset of A compartments that are enriched for PRC2 binding. To which extent the latter could represent I compartments, similar to the ones described in activated B cells (29) remains to determined. Loss of H1 either in B or T lymphocytes was also shown to induce a profound reprogramming of epigenetic states with an expansion of H3K36me2 deposition at the expense of H3K27me3, suggesting that H1 binding plays an active role in balancing these modifications (203). Deletion of *H1c* and *H1e* in murine GC B cells conferred enhanced fitness and self-renewal, while at the molecular level inducing large-scale, but focal, chromatic decompaction and de-repression of stemness signature gene (202). Accordingly, Hi-C analysis revealed thousands of B-to-A compartment switches, which mostly represented expansion of A compartments, coinciding with spreading of H3K36me2. The above studies unravel novel and intricate functions of H1 linker histones in regulating 3D chromatin organization, epigenetic states and transcriptional activity that go beyond B/T cell biology (204).

### Nuclear Lamina

Nuclear Lamina (NL) serves as a scaffolding deck for heterochromatin, shaping chromatin compartmentalization and regulating gene silencing (205). Alterations in the NL compartment have been also reported upon lymphocyte activation. As shown by early electron microscopy studies, resting lymphocyte present a compact heterochromatin at the nuclear periphery which dissociates upon activation and gene expression (90, 206–208).

In lymphocytes, tethering of gene loci on NL and their repositioning towards the nuclear center is considered as a safety mechanism to prevent lymphocyte premature activation or recombination (209–211). For example, localization of *Ig* loci at the NL in pre-pro-B cells, prevents access by RAG proteins, impeding premature V(D)J recombination, while their release from the NL in pro-B cells constitutes a major event enabling B cell fate and proper BCR expression (209). Moreover, while proximal V, D and J sequences are released from the NL in pro-B cells, distal V segments remain lamin-associated, ensuring the spatiotemporal control of antibody repertoire diversity. Relocalization from NL has also been shown in the context of T cell activation. Upon TCR activation, T cell-specific genes and their enhancers are repositioned from LAD-associated subcompartments, to TAD-proximal subcompartments, although they still remain in a proximity to the nuclear periphery <0.6 µm (210). This “constrained release” mechanism contributes to a fast transcriptional response upon T cell activation, that is lost when specific T cell genes and enhancers relocate towards the permissive perinuclear domains. Another example of repositioning from the nuclear periphery in T cells was shown for the BCL11B genome organizer (211). During thymic T cell development in DN2 cells, a long non-coding RNA named thymocyte differentiation factor (*ThymoD*) promotes the demethylation of CTCF bound sites and activates cohesin-dependent looping to juxtapose the *Bcl11b* enhancer and promoter into a single-loop domain leading to *Bcl11b* expression (211).

Despite the strong association of nuclear periphery with heterochromatin and gene silencing, there are a few relevant and noteworthy exceptions of gene expression. 3D-immuno DNA/RNA FISH experiments in plasma cells revealed that the transcribed *Igh*, *k* and *j* genes (located on chromosomes 12, 6 and 5 respectively), are spatially clustered at the nuclear periphery with RNA polymerase II transcription factories (212). These *Ig* genes are not localized to the NL but close to nuclear pore and reticulum endoplasmic, in support of the previously proposed ‘gene gating theory’ (213). This process has been suggested to facilitate Ig mRNA export and maximize the antibody production process. Active transcription at the nuclear periphery of lymphocytes was also reported for immune-specific microRNA genes (*miR-181a1b1*, *miR-181a2b2*, *miR-181c*, *miR-142*, *miR-146a*, *miR-17-92* and *miR-155*) during T cell development (93). These genes are located within the constitutive inter-LADs (ciLADs), while their peripheral position is conserved throughout development (from ESCs to thymocytes, CD4^+^ and TH cells). Moreover, ChIP-seq analysis showed that microRNA genes are occupied by NUP153/93 and DROSHA proteins, suggesting spatial links among transcription, post-transcriptional processing and nuclear export (93).

The importance of nuclear lamina for LAD organization together with the strong association between LADs and B compartments, argue for the potential function of NL for proper chromatin compartmentalization. To address this question, a recent study deleted Lamin B receptor (LBR^-/-^) in thymocytes and tracked changes in nuclear organization by imaging and compartmentalization by Hi-C analysis (214). As expected, LBR^-/-^ thymocytes presented an inverted nuclear architecture with heterochromatin localized in the nuclear center while euchromatin pushed towards the periphery. However, despite this striking change in subnuclear organization, the authors detected only moderate changes in compartmentalization and TAD organization. This suggests that NL is critical for the peripheral positioning of heterochromatic regions but not involved in the spatial segregation between heterochromatin and euchromatin (B and A compartments).

Taken together, the abovementioned reports support that during lymphocyte development and activation several dynamic chromatin changes take place at the NL compartment, controlling the expression of immune-related genes. Lymphocyte nuclear periphery is not exclusively linked to gene repression, but can also function as a permissive microenvironment, that can host either accessible chromatin regions and/or active genes that are important for lymphocyte development and adaptive immune responses.

## CHAPTER V: Alterations of the 3D Chromatin Organization Upon Lymphoid Transformation

Alterations that affect the function or levels of critical TFs and epigenetic modulators are well-appreciated drivers of lymphoid transformation by inducing a global dysregulation of the transcriptional program and epigenetic landscape (215, 216). Increasing evidence supports that perturbations of structural proteins involved in 3D chromatin organization might also play critical roles in lymphoid malignancies (217). The development of advanced imaging and 3C techniques over the past years combined with elegant genetic models allowed for a better understanding of the extent of 3D chromatin dysregulation during lymphoid malignancies and its potential role in driving transformation (**Table 1**).

**Table 1:** Overview of 3D chromatin alterations in lymphoid malignancies.

Lymphoid malignancies	Structural rearrangments and mutations	3D chromatin layers alterations			Molecular mechanisms	References	
		Compartments A/B and LADs	TAD (length, activity, insulation)	Loops			
B-ALL and DLBCL	–	Lamin A/C downregulation	–	–	Promoter hypermethylation	(218, 219)	**Nuclear lamina**
CLL and GC-derived lymphomas	–	Lamin B1 downregulation	–	–	Unknown (no promoter hypermethylation). Post-transcriptional?	(220)	
T-ALL	Change in CTCF binding site accessibility	10% of switches	TAD boundaries insulation modulation in correlation with CTCF binding and intra-TAD activity changes	Notch-dependent E-P loops	*MYC* Proto-oncogene activation (TAD Fusion at Myc locus due to the loss of CTCF-mediated insulation)	(221)	**CTCF and cohesin**
	CTCF binding alterations	–	–	CTCF-mediated loops	*NOTCH*-induced CTCF binding gain associated with enhancer activities	(222)	
B-ALL	*CTCF* deletion	Not affected	TAD boundaries disruption	E-P loops	*MYC* Proto-oncogene activation	(223)	
	*CTCF* downregulation and mutations	–	Reduction of TAD boundaries insulation	–	Aberrant E-P looping upon TAD boundary insulation reduction	(224)	
DLBCL	*SMC3* mutations	–	Reduction of TAD boundaries insulation and increased inter-TAD activity	Loss of E-P loops	loss in E-P connectivity at lymphoma-associated TSG	(225)	
MM	–	–	Increase of TAD boundaries insulation and changes in intra-TAD activity	–	Increase CTCF peaks in NSD2^High^ cells contribute to the weakening of compartment structure	(226)	
DLBCL	Histone 1	A to B switches	No change in TAD boundaries insulation but gain of intra-TAD activity	Gain of E-P loops for stem cell genes	Genome-wide decompaction allowing for the abnormal expression of stem cell genes in GC B-cells	(227)	**Epigenetic modifiers**
DLBCL and FL	Ezh2^Y646X^	–	–	–	TSG silencing in inactive TADs	(228)	
T-ALL	BCL11B-TLX3 among others	Minor switches (1.5%)	–	–	*BCL11B* enhancer hijacking	(113)	**Enhancer hijacking**
B-ALL	TCF3-HLF	–	–	–	*MYC* enhancer hijacking	(229)	
BCP-ALL	FLT3 deletion	–	Loss of TAD boundaries	–	Switch of *FLT3* enhancer	(113)	
Ph+ B-ALL	*GATA3* germline variant	–	–	–	*CRLF2* enhancer hijacking	(113)	
MM	IgH-CCND1	–	–	–	Epigenomic translocation of H3K4me3 broad domains following super-enhancer hijacking	(230)	
CLL/MCL	–	A to B switches in CLL and B to A switches in MCL	–	–	Compartment switch leading to the abrogation/increase of interactions and RE in a disease-specific manner	(29)	**Others**
T-ALL	–	–	–	–	*TAL1* / *LMO2* Proto-oncogene activation	(231)	
	–	–	–	Loss of E-P loops	*TAL1* Proto-oncogene activation	(232)	
DLBCL	IgH-BCL6	–	*de novo* TAD and TAD boundaries loss	Loop strength unchanged	Gain of TAD structure of cancer-related genes	(40)	
MCL	IgH-CCND1	–	–	–	Perinucleolar relocalization of IgH-CCND1 allele	(233)	
	–	–	–	–	*NOTCH*-mediated E-P repositionning and formation of 3D-Cliques	(177)	
MM	–	A to B (8%) switches and B to A (24%) switches	25% increase in *de novo*TADs (partitioning) and overlap with CNV breakpoints	–	CNV-mediated disruption of TAD boundaries affecting MM-related pathways and key genes expression.	(234)	

B-ALL, B-cell Acute Lymphoblastic Leukemia; DLBCL, Diffuse Large B-cell Lymphomas; CLL, Chronic Lymphocytic Leukemia; MCL, Mantle Cell Lymphoma; T-ALL, T-cell Acute Lymphoblastic Leukemia; MM, Multiple Myeloma; FL, Follicular Lymphoma; BCP-ALL, B-Cell Precursor Acute Lymphoblastic Leukemia; Ph+ B-ALL, Philadelphia chromosome-positive ALL; TAD, Topologically Associating Domain; CNV, Copy Number Variation; E-P, Enhancer-Promoter; TSG, Tumor Suppressor Gene; RE, Regulatory Element.

### Lamin Dysregulation

One of the first nuclear organizers that were discovered to promote lymphoid transformation were Lamin A/C. Downregulation of type A Lamin genes by hypermethylation of Lamin A/C promoters occurs in about 20% of B-cell Acute Lymphoblastic Leukemia (B-ALL) and 35% of Diffuse Large B-cell Lymphomas (DLBCL) patients and are associated to poor prognosis (218, 219). Although the loss of A/C lamin was hypothesized to contribute to genomic instability and aneuploidy by preventing proper cytokinesis (235), defects on 3D chromatin organization cannot be excluded. Lamin B1, which maintains the *Igh* V genomic segments within the repressive heterochromatin in naive B cells, has also been shown to be involved in B-cell malignancies (220). Its transient downregulation has been reported to be necessary for the GC reaction and more particularly for *Igh* V transcription and somatic hypermutation (SHM) upon antigen encounter. In comparison to normal human reactive lymph nodes, Lamin B1 expression has been documented to be further decreased and permanently locked in primary GC derived B-cell lymphoma and in transformed Follicular Lymphoma (FL). Together, these reports support a role for lamins and LADs dysregulation in the initiation and the progression of lymphoid malignancies beyond genomic instability.

### Compartment Switches

A/B-compartment switching has been identified in T and B cell malignancies, albeit the functional and biological consequences of these alterations remain to be determined, in a cell-specific and context-dependent manner. A recent study comparing Chronic Lymphocytic Leukemia (CLL) and Mantle Cell Lymphoma (MCL) patient samples to normal B cells reported that about 25% of the genome undergoes compartmental changes (29). These switches were mostly associated to a global inactivation (A to B) in CLL and activation (B to A) in MCL, suggesting that while similar cellular transformation mechanisms might be in place for these neoplasms supposedly originating from memory and naive B cells, disease-specific 3D chromatin topology alterations might be uncovered (29). When comparing CLL to naive B cells, the *EBF1* locus was associated with a shift from A to I compartment along with a drastic loss of enhancer activity and weakening of E-P interaction, consistent with the low expression of the *EBF1* gene as a diagnostic marker in CLL. A similar study comparing T-cell Acute Lymphoblastic Leukemia (T-ALL) genome to normal peripheral T-cells revealed that about 10% of the genome experienced compartmental changes which were subtype-specific (221).

### Changes in TAD Activity and Boundary Insulation

Common TAD alterations in cancer include TAD fusions or splits due to the loss or *de novo* establishment of TAD boundaries, respectively (236). These changes have been associated with downregulation of Tumor Suppressor Genes (TSG) or overexpression of oncogenes within TADs. About 10% of TAD boundaries changed upon T-cell transformation to T-ALL, with more than half of these changes associated with altered CTCF binding (221). At the global level, this study demonstrated a correlation between increased intra-TAD activity and transcriptional upregulation as well as higher boundary insulation enriched with CTCF and Notch binding. Conversely, weakening of TAD boundaries and downregulation of proximal genes was recently described in hyper-diploid B-ALL patients which usually present lower CTCF expression (224).

Using a low-input Hi-C method, Diaz et al. were able to profile chromatin interactions in primary DLBCL cells in comparison to peripheral B cells. This analysis uncovered >600 regions with altered interaction patterns and 6% of these corresponded to *de novo* patients-specific TADs. Furthermore, some of these neo-TADs were located around cancer related genes such as *TP63* or *TPRG1* in the vicinity of *BCL6* locus (40) although both the driving mechanisms and functional consequences of these reorganization remain to be tested. Similarly, the Hi-C analysis in DLBCL cell lines with gain of function Ezh2 mutation (Ezh2^Y646F^) could not identify major topological changes compared to Ezh2^WT^ cells but only focal effects on selected TADs (228). Increased deposition of H3K27me3 occurred predominantly around regions that were already decorated by this mark, arguing for a possible spreading mechanism rather than *de novo* establishment. Along with increased levels of H3K27me3, the authors identified transcriptional downregulation of multiple TSG such as *FOXO3* and *ARMC2* within “inactive” TADs due to loss of promoter interactions (228).

### Enhancer-Promoter Rewiring

3D chromatin changes that directly affect E-P communication of tumor suppressor genes or proto-oncogenes have been extensively described in hematological malignancies (236, 237). The MYC oncogene is a critical regulator of NOTCH1-mediated T-ALL. A long-range interaction between *Myc* promoter and its distal (~1.4 Mb) Notch1-bound enhancer (N-Me) occurs transiently during T-cell development, specifically at DN3 and DN4 stage, to support Notch-Myc driven growth. More than 60% of T-ALL patients show constitutive activation of the NOTCH pathway and aberrant upregulation of Myc expression, partly mediated through the re-establishment of N-Me/Myc contact, as confirmed by conditional deletion of N-Me in mice (238). Another study in T-ALL, identified a recurrent TAD fusion around the *Myc* locus along with a major increase in inter-TAD interactions upon the loss of Notch/CTCF-mediated insulation as shown by 4C-seq and functionally validated by 3D DNA-FISH (221). The role of NOTCH1 in aberrant 3D chromatin reorganization has been also reported in B cell lymphoma, where NOTCH1 mediates spatial clusters of long-range E-P interactions forming hyperconnected “3D cliques”, which include crucial protooncogenes (177).

### Mechanisms for 3D Reorganization in Cancer

Genetic or epigenetic alterations that affect the function or binding of genome organizers is the most common mechanism which induces local or global topological changes during malignant transformation. Heterozygous, loss-of-function mutations of key architectural factors, such as CTCF and various subunits of the cohesin complex, are frequently detected in myeloid (239–241) and lymphoblastic leukemia (242, 243). Initially it was proposed that cohesinopathies, could be involved in tumorigenesis by the induction of genomic instability (244). While this hypothesis remains plausible, most of blood malignancies demonstrate limited aneuploidy arguing for additional mechanisms.

A recent study, using a conditional knock out (cKO) mouse model showed that haploinsufficiency of the cohesion subunit Smc3 in GC B cells, leads to GC hyperplasia and impairs plasma cell differentiation (225). Hi-C analysis revealed an overall reduction of TAD boundaries insulation resulting in increased inter-TAD associations. More importantly, there was a significant reduction of intra-TAD interactions in Smc3-haploinsufficient GCs, which correlated with transcriptional downregulation of implicated genes. The most dramatic loss in connectivity occurred around enhancers and promoters, especially around lymphoma-associated TSG, such *Dusp4*, *Zeb2* and others.

In addition to genetic alterations of architectural factors, epigenetic changes might also affect binding and/or function of these proteins and perturb loop extrusion. Indeed, CTCF binding is sensitive to cytosine methylation within the CTCF-binding elements (CBE) (160). Given that global methylation alterations frequently occur in T and B-cell malignancies due to *TET2*, *DNMT3A* and *IDH2* mutations (245–247), it is reasonable to expect changes in CTCF binding and thus, in TAD boundary insulation. Mutations on CBE elements may also affect CTCF binding and local chromatin conformation (162) and could possibly function as cancer drivers (248). Surprisingly, loss of CTCF binding at specific TAD boundaries and CBE in T-ALL is neither associated with somatic mutations nor is accompanied by increased DNA methylation but rather with a localized reduced chromatin accessibility (221). This new paradigm of alteration of looping machinery organizer remains to be further investigated in other immune related diseases and malignancies as well.

Chromosomal rearrangements originating from double strand breaks (DSBs) are frequent in lymphoid malignancies and do not constitute a stochastic process, as intrachromosomal segments are more frequently targeted compared to interchromosomal ones in B cells (249, 250). Integration of Hi-C profiling and DSB induction by etoposide in B cells demonstrated that TAD boundaries are enriched for CTCF/cohesin while topoisomerase II complexes are hotspots for genomic rearrangements (251). The chimeric TF TCF3-HLF, which confers treatment resistance in ALL was recently shown to act as a pioneer factor that aberrantly activates a distal *Myc* enhancer and mediates interaction with the its promoter and therefore its overexpression (229). While this chimeric TF seems to interact with ETS factors to regulate enhancer function, it was also shown to physically form a complex with multiple chromatin organizers such as CTCF and YY1 as well as with the histone acetyltransferase p300 (EP300). Indeed, TCF3-HLF deletion leads to a reduction of EP300 mediated H3K27ac deposition at TCF3-HLF binding sites. In agreement, *in vivo* treatment with JQ1 and A-485 inhibitors caused reduced BRD recruitment on enhancers and downregulation of *Myc* expression, indicating p300 or BRD proteins as potential therapeutic target for ALL.

Finally, deletions at 13q12.2 in B-Cell Precursor Acute Lymphoblastic Leukemia (BCP-ALL) lead to the loss of TAD boundaries and gain/rewiring of E-P loops sustaining the expression of the common leukemia driver FLT3 (252). Similarly, *de novo* long-range interactions enabling the expression of the protooncogenes *TAL1* and *LMO2* (231) have been documented in T-ALL, posing the disruption of insulated neighborhoods as a new paradigm in lymphoid transformation.

### Reversibility of Chromatin Alterations and Therapeutic Strategies

Chromatin conformation techniques have been proposed to serve as novel diagnostic tools by looking at the emergence of translocations, copy number alterations (CNA) and new regulatory loops or subtypes within human B-cell malignancies (253). Theoretically, DNMTi (5-azacytidine and decitabine) treatment for patients presenting a hypermethylated genome along with *TET*, *IDH1/2* or *DNMT3A* mutations, could have therapeutic potential by restoring CTCF binding to CBE. While this treatment is increasingly used in clinic (254), the effects on CTCF binding and 3D chromatin organization have widely not been investigated. However, a recent study in T-ALL cell lines treated with 5-azacytidine showed no restoration of CTCF binding, challenging this therapeutic opportunity (221).

Clinical trials assessing another inhibitor targeting the catalytic activity of Ezh2 (255), was sufficient to re-activate the expression of multiple tumor suppressor genes (such as *FOXO3*, *SESN1*, and *ARMC2*) and restore the connectivity of the respective TADs in Ezh2^Y646F^ mutated B-cell lymphoma cells, without inducing other changes of the chromatin compartmentalization into TADs (228). Finally, treatment of T-ALL cell lines with NOTCH1 inhibitor *γ*-secretase (γ-SI) led to loss of H3K27ac from select (sensitive) enhancers, without affecting intra-TAD activity nor TAD boundary insulation. However, treatments with the CDK7 inhibitor THZ1 was able to reduce the activity of *Myc* distal enhancers as well as their interaction with *Myc* promoter and restore the original TAD structure within the *Myc* locus, which remain unaltered upon *γ*SI treatment (221).

## Discussion

Over the last decades, multiples studies have shed light into the principles, mechanisms and biological significance of 3D chromatin reorganization occurring upon lymphopoiesis. Although these studies offered snapshots of specific stages and transitions, many pieces in the 3D puzzle of lymphopoiesis are still missing due to technical difficulties to capture and/or characterize transient and dynamic subpopulations.

How is 3D genomic architecture reorganized throughout lymphopoiesis and upon immune response and how does it associate with transcriptional changes? Application and further improvement of single-cell multi-omics technologies (such as scHi-C (115, 256), HiCAR (257), GAM (258), scRNA/ATAC (259) etc.) at high-resolution, will enable the construction of complete 3D lymphopoietic molecular roadmaps. It will provide a better understanding of the temporal interconnections between 3D organization, chromatin state, transcription and cell fate. This will also enable the precise 3D chromatin mapping of specific developmental stages that have not been investigated so far (e.g. memory B cells). As recently described for CTCF loss in murine B cells, special attention should be paid to cytokine-mediated ex vivo activation, as an alternative to the in vivo characterization and isolation. As shown in B cells, CD40 activation mimics the in vivo CTCF loss sensitivity on the contrary to IL-4/LPS treatment (165).

The recent development of CRISPR-(d)Cas9 (260), Degron (127) and super-resolution live imaging technologies allowed inducible spatiotemporal perturbation of cellular processes (including transcription and cell cycle modulation) and genome organizers function. Using such approaches, will enable to decipher in a more definitive and quantitative manner the extent to which 3D chromatin organization and transcriptional activity are functionally interconnected during lymphopoiesis. As we discussed above, a large number of 3D organization players have been suggested to mediate E-P loops and to be involved in compartment, hyperconnected TAD or hubs. However, most of the studies provide associations and not direct experimental evidence for the architectural roles of these factors and the underlying mechanisms.

Which factors and processes are critical for building or maintaining 3D chromatin organization at different stages of B/T lymphopoiesis? An answer to this question might be provided through the involvement of the non-coding RNAs, which are increasingly appreciated as chromatin mediators in various systems (261). Given that a large number of non-coding RNAs are expressed in the immune system and specifically during lymphopoiesis (211, 262), they could possibly be considered as good candidates controlling local chromatin topology and distal interactions. Hence, further investigations towards this direction should be conducted. Although the phase separation mechanism has only recently been introduced in other cell types, 3D nuclear architecture studies in this direction, during lymphocyte development and activation could shed more lights on understanding the regulatory landscapes and their correlation with gene expression alterations and diseases (263, 264). Identifying the mediators and the principles of 3D chromatin reorganization that ensures proper lymphocyte development and differentiation, is critical to determine their potential alterations in lymphoid malignancies. Targeting specific architectural dysregulations (such as enhancer hijacking, cliques, etc.) that take place upon lymphoid transformations, might in the future open avenues to the development of novel therapeutic strategies.

## Author Contributions

LS, ES, and EA conceptualized the manuscript. LS drafted all B-cell relevant parts and the tumorigenesis chapter. ES drafted all T-cell relevant parts. EA supervised, coordinated and wrote the final manuscript. LS and ES contributed equally. EA is the corresponding author. All authors contributed to the article and approved the submitted version.

## Conflict of Interest

The authors declare that the research was conducted in the absence of any commercial or financial relationships that could be construed as a potential conflict of interest.
